# New Cinchona Oximes Evaluated as Reactivators of Acetylcholinesterase and Butyrylcholinesterase Inhibited by Organophosphorus Compounds

**DOI:** 10.3390/molecules22071234

**Published:** 2017-07-22

**Authors:** Maja Katalinić, Antonio Zandona, Alma Ramić, Tamara Zorbaz, Ines Primožič, Zrinka Kovarik

**Affiliations:** 1 Institute for Medical Research and Occupational Health, POB 291, HR-10001 Zagreb, Croatia; mkatalinic@imi.hr (M.K.); azandona@imi.hr (A.Z.); tzorbaz@imi.hr (T.Z.); 2 Department of Chemistry, Faculty of Science, University of Zagreb, HR-10001 Zagreb, Croatia; alma.ramic@gmail.com

**Keywords:** nerve agents, pesticides, alkaloids, cytotoxicity, reversible inhibition

## Abstract

For the last six decades, researchers have been focused on finding efficient reactivators of organophosphorus compound (OP)-inhibited acetylcholinesterase (AChE) and butyrylcholinesterase (BChE). In this study, we have focused our research on a new oxime scaffold based on the Cinchona structure since it was proven to fit the cholinesterases active site and reversibly inhibit their activity. Three Cinchona oximes (C1, C2, and C3), derivatives of the 9-oxocinchonidine, were synthesized and investigated in reactivation of various OP-inhibited AChE and BChE. As the results showed, the tested oximes were more efficient in the reactivation of BChE and they reactivated enzyme activity to up to 70% with reactivation rates similar to known pyridinium oximes used as antidotes in medical practice today. Furthermore, the oximes showed selectivity towards binding to the BChE active site and the determined enzyme-oxime dissociation constants supported work on the future development of inhibitors in other targeted studies (e.g., in treatment of neurodegenerative disease). Also, we monitored the cytotoxic effect of Cinchona oximes on two cell lines Hep G2 and SH-SY5Y to determine the possible limits for in vivo application. The cytotoxicity results support future studies of these compounds as long as their biological activity is targeted in the lower micromolar range.

## 1. Introduction

Organophosphorus compounds (OPs), commonly used as pesticides, but also as an insidious threat in terrorism, are covalent inhibitors of the enzymes acetylcholinesterase (AChE, EC 3.1.1.7) and butyrylcholinesterase (BChE, EC 3.1.1.8) phosphylating their active center serine. The inhibition in vivo triggers a cholinergic crisis, resulting from the accumulation of acetylcholine and overstimulation of receptors in synapses. Therefore, human exposure to these hazardous compounds results in acute poisoning manifested by salivation, tremors, respiratory paralysis, hypotension, and, with more extreme exposure, death [1].

Efficacy of the oxime therapy, typically 2-PAM, obidoxime or HI-6, combined with atropine and an anticonvulsant, is still limited [2,3]. Though the symptoms of poisoning will be somewhat attenuated by atropine and an anticonvulsant, the applied oximes will not be equally effective in reactivating AChE inhibited by several nerve agents [1,2,3], and with that the cholinergic crisis will persist leading to the unwanted consequences. In addition, inhibitory OPs remain in different body compartments permitting redistribution into the plasma, where they are capable of inhibiting or re-inhibiting active AChE at target sites [4]. The effectiveness of oxime-assisted reactivation is primarily attributed to the nucleophilic displacement rate of organophosphates, but efficiency varies with the structure of the bound organophosphate [5,6,7]. This fact has led to the synthesis and investigation of numerous oximes in the past sixty years. One direction for an efficiency improvement has led to the design of advanced oximes with a peripheral anionic site (PAS) moiety for anchoring the oxime molecule at the PAS [8,9]. Recent studies have also focused on the development of uncharged species to improve delivery to the central nervous system [9,10,11,12].

**Figure 1:**
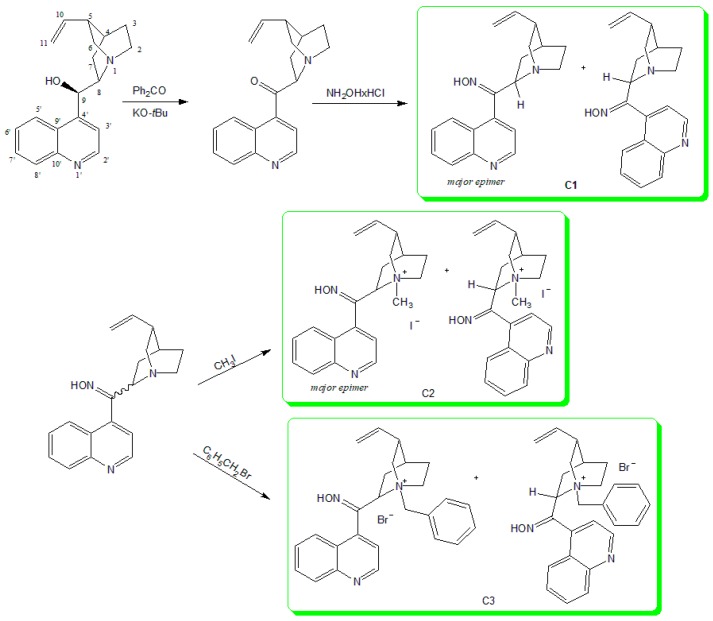
Synthesis of Cinchona derivatives starting from cinchonidine.

This paper reports on the synthesis of three derivatives of Cinchona oxime compounds. Some derivatives of cinchonidine have been identified as inhibitors of cholinesterases fitting their active site [13,14]. Therefore, we found them interesting for oxime moiety addition and their evaluation as reactivators of human AChE (hAChE) and BChE (hBChE) inhibited by OPs. To test this scaffold’s properties in the design of potential antidotes, we synthesized and prepared three oximes (C1, C2 and C3), bearing the oxime moiety in the central linker and differing in the side chain of the quinuclidine ring (Figure 1). We evaluated their reactivation potential in VX, sarin, cyclosarin, tabun and paraoxon poisoning as well as determined their affinity for reversible binding to AChE and BChE. Furthermore, we also tested their cytotoxicity on two cell lines, hepatocytes (Hep G2) and neurones (SH-SY5Y), to assess their potential to be used in in vivo application.

## 2. Results and Discussion

Cinchonidin-9-one was prepared by oxidizing cinchonidine with potassium *t*-butoxide and benzophenone in toluene [15]. The reaction of ketone with hydroxylamine hydrochloride in ethanol resulted in the Cinhona 9-oxime (C1) as an off-white solid in good yield. The nitrogen atom of the quinuclidine part of the molecule was quaternized with methyl iodide (C2) or benzyl bromide (C3) in dry acetone (Figure 1). All compounds were prepared in good yields and were characterized by standard analytical spectroscopy methods (1D and 2D NMR, IR, MS). Robins studied tautomerism of cinchonidine-9-one in polar solvents and concluded that it undergoes mutarotation forming a mixture of 8*S*- and 8*R*-epimers, which can be seen in the ^13^C-NMR spectrum [15]. During the synthesis of our compounds, in reaction with hydroxylamine in ethanol, epimerization at position eight of the quinuclidine ring occurred as expected, with the ratio of epimers 8*R*:8*S* = 70:30 for C1. Furthermore, epimerization was observed during the synthesis of C2 (8*R*:8*S* = 60:40) and C3 (8*R*:8*S* = 50:50). Thus, upon resting in a polar solution, additional resonances in ^1^H- and ^13^C-NMR spectra of all compounds were observed. The same effect was detected for oximes due to the possibility of forming *syn-* and *anti*-isomers. To confirm these findings, quantum chemical for all possible isomers of Cinchona 9-ketone and 9-ketoximes were carried out. For each compound geometry optimizations and harmonic frequency calculations using density functional theory (B3LYP/6-311++G(d,p)) were performed. All harmonic frequencies were real confirming that the obtained structures are local minima. Subsequently, magnetic isotropic shielding was calculated at the same level of the theory. NMR spectra were estimated and compared to the experimentally obtained one. Observed differences in the set of NMR data for each C8-epimer of compound C1 which are separated chromatographically, were compared to the calculated values. The most useful differences detected in experimentally obtained data were assigned to carbon atoms C2 and C6, (Materials and Methods and Supplemental Materials) in ^13^C-NMR spectra, and to hydrogen atom H10 in ^1^H-NMR spectra (Materials and Methods and Supplemental Materials). DFT calculation enabled the assignation of spectra in respect of C8-tautomers by comparing relative values of chemical shifts for each atom in the present species, Tables S1–S3.

**Figure 2:**
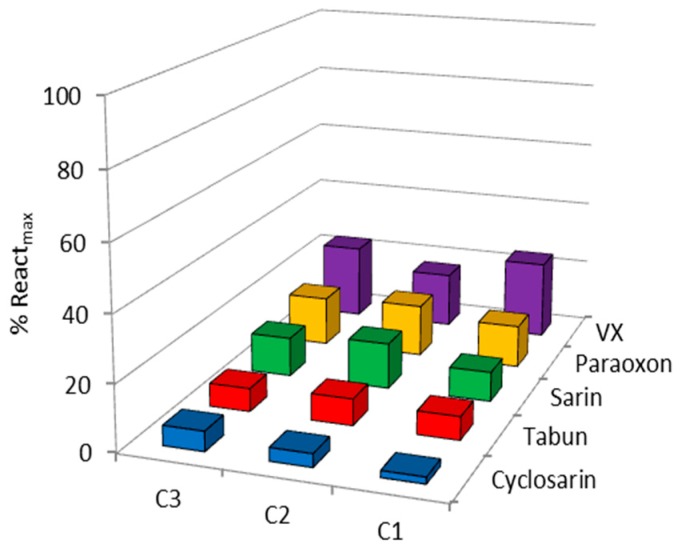
Screening of organophosphorus compound (OP)-inhibited human acetylcholinesterase (hAChE) reactivation by 0.1 mM oximes C1, C2 and C3 at 25 °C. Results in terms of the maximal obtained reactivation percentage within 23 h are presented as a mean of two to three experiments (experimental deviation was less than 10%).

**Figure 3:**
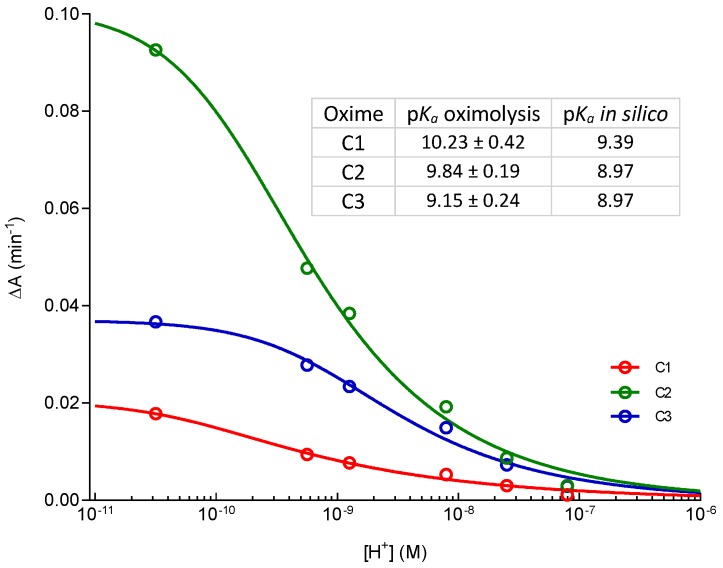
[H^+^]-dependence of oximolysis rate for C1, C2 and C3 oximes (0.1 mM) and their in vitro and in silico determined p*K*_a_ values. Acid dissociation constant (*K*_a_) of the oxime group was determined by measuring the degradation of substrate acetylthiocholine (1 mM) by 100 µM oxime at 412 nm and in pH range 4.4–11.3 and was calculated using the Equation (1) (see Section 3.4).

Oximes were profiled in the reactivation of hAChE inhibited by VX, sarin, cyclosarin, tabun and paraoxon using 0.1 mM oximes. This concentration is widely used in the screening of oximes’ reactivation potency and by using this concentration we were able to directly compare our results with published literature data [9,16,17]. Also, this concentration is considered applicable to humans [1,2]. The obtained results for the hAChE reactivation are summarized in Figure 2. As our results indicate, the maximal achieved reactivation was less than 25%, and the overall low hAChE reactivation potency was irrespective of the structure of an oxime or a bound organophosphorus compound, which maybe implies low binding affinity to the OP–enzyme complex due to spatial constraints within the narrow active site gorge of hAChE. These results also indicate that the positioning of these three compounds is unfavorable for reactivation within the hAChE’s active site. Moreover, in silico and in vitro determined high p*K*_a_ values (Figure 3) of the oxime group could be correlated with lower nucleophilicity of Cinchona oximes, since expected concentration of the more nucleophilic oximate ion at pH 7.4 is low (about 2–3% of total compound concentration).

**Figure 4:**
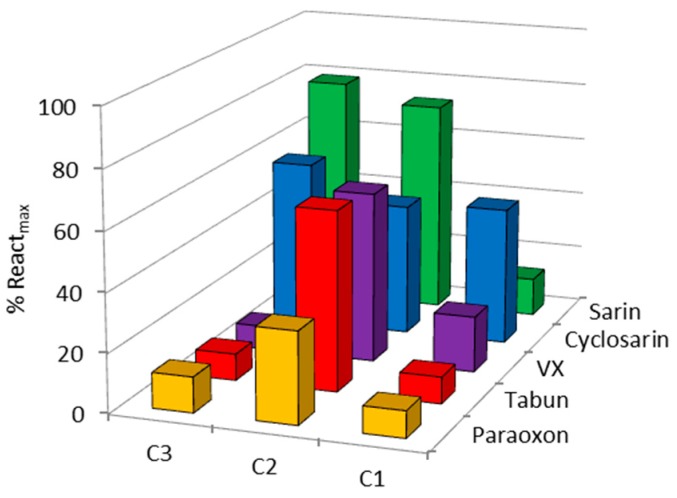
Screening of OP-inhibited human butyrylcholinesterase (hBChE) reactivation by 0.1 mM oximes C1, C2 and 0.05 mM C3 at 25 °C. Results in terms of maximal obtained reactivation percentage within 22–24 h are presented as a mean of two to three experiments (experimental deviation was less than 10%).

In the case of hBChE reactivation, the results were more promising; the maximal obtained reactivation rose up to 70% (Figure 4 and Figure S1). This was especially pronounced in the reactivation of sarin-inhibited BChE by oximes C2 and C3. The observed reactivation rate constants, *k*_obs_, were also evaluated where possible, in other words, where the observed reactivation exceeded 40% (Figure S1). The results are summarized in Table 1. As can be seen, the absence of a permanent charge in C1 did not benefit the reactivation and yielded the weakest results. This is contrary to other studies that showed a potential of the uncharged oximes in hBChE reactivation [8,16]. On the other hand, oxime C2 stood out as the most active against all of the OPs. Though the calculated rate constants should be much higher if one considers future application of this oxime in therapy/pretreatment, such a C2 Cinchona scaffold seems to be promising for the design of a long-desired universal reactivator for hBChE reactivation. According to recent results on hBChE reactivation, several pyridinium, imidazolium and even non-pyridinium oximes were pointed out as promising but still showed selectivity towards different OPs [8,17,18,19,20,21]. Although proper investigation of oximes as reactivators requires the determination of time- and concentration-dependent reactivation to give insight into reactivity and affinity, we were not able to test a wider oxime concentration range due to the observed strong affinity of uninhibited hBChE for these oximes (especially seen for oxime C3 wherefore 0.05 mM was used in reactivation tests). However, we suppose that the additional modification of the Cinchona 9-oxime structure tuning the affinity and efficacy (e.g., towards lowering the p*K*_a_ of the oxime group) could result in an even better reactivation outcome.

**Figure 5:**
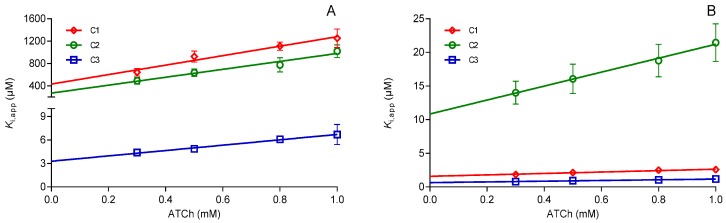
Reversible inhibition of (**A**) hAChE and (**B**) hBChE by the tested oximes. The graphs present Hunter-Downs plots with the average of the experimentally obtained values from at least three experiments. *K*_i_ presenting the Y-intercept was studied in 0.3–1.0 mM substrate acetylthiocholine (ATCh) concentration range.

The observed effect prompted us to evaluate these oximes as reversible inhibitors of hAChE and hBChE. The obtained results are summarized in Table 2 and presented as Hunter-Downs plots in Figure 5. The oximes inhibited both cholinesterases in micromolar range but showed a strong preference for binding to hBChE. This confirmed previous studies on similar compounds [13,14] but also indicated that the addition of the oxime moiety did not influence their ability to bind to the cholinesterases active site. Moreover, the evaluated dissociation inhibition constants (*K*_i_) were higher than those published for pyridinium oximes and hAChE [22,23,24], and were 10–100-fold lower for pyridinium and imidazolium oximes and hBChE [17,25].

The experimental data presented in terms of the Hunter-Downs plot (Figure 5) enabled the estimation of a binding mode for the tested oximes. In such a plot, possible competitive or non-competitive reversible inhibition is indicated by a slope value: if inhibition is competitive, the slope of the plot is >> 0; if non-competitive, the slope is zero [26]. In the case of cholinesterases, and due to the presence of the catalytic and the allosteric part of the specific active site structure, a mixed type inhibition is possible too. The type of inhibition could be confirmed by the evaluated enzyme-substrate dissociation constant *K*_S_ (X-intercept in the Hunter-Downs plot) which corresponds to the Michaelis constant (*K*_M_). Values closer to the *K*_M_ (0.05 mM) will indicate a competitive type of reversible inhibition, while those higher than 10 mM (*K*_SS_, enzyme-substrate dissociation constant for complex formed at the allosteric site) indicate a non-competitive one [27]. Therefore, looking at the slopes (Figure 5) and *K*_S_ values determined for oximes C1, C2, and C3 (ranging from 0.4–0.96 mM for hAChE and 1.05–1.5 mM for hBChE), we can say that the predominant type is mixed inhibition marked by binding at both the catalytic and the allosteric part of the cholinesterases active site. The strongest binding to both enzymes was observed for oxime C3, which had additional benzyl group attached to the quinuclidine ring, as was estimated from the reactivation experiments. This oxime also showed the lowest selectivity between the two enzymes. Interestingly, oxime C1 showed to be the most selective in binding and inhibited BChE about 300-fold more strongly than hAChE. Such a preference for binding compounds like C1 without permanent charge could benefit the development of selective inhibitors for condition where such inhibition is a proposed way of treatment as it is the case with neurodegenerative diseases [28,29,30]. Hence, the C1 scaffold could be used for structure refinement in the future.

**Figure 6:**
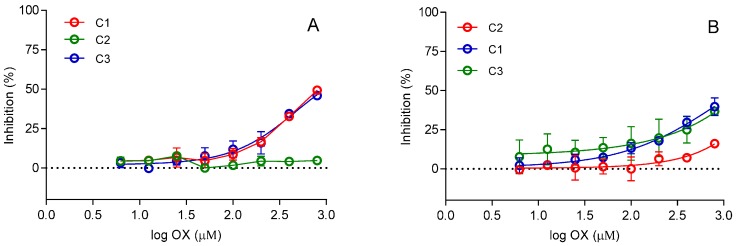
Cytotoxicity of oximes C1, C2, and C3 on two cell lines (**A**) HepG2, human Caucasian hepatocyte carcinoma, epithelial; (**B**) SH-SY5Y human neuroblastoma cell line.

Nonetheless, before being considered for any in vivo application, the compounds need to pass rigorous test spanning, much more than in in vitro kinetic studies. To profile the potential of this Cinchona scaffold for further development, we screened the cytotoxicity of oximes C1, C2, and C3 up to 800 µM on two selected cell types: hepatocytes (Hep G2) and neurones (SH-SY5Y). The results form of IC_50_ graphs are presented in Figure 6. As can be seen, hepatocytes showed to be more sensitive to these oximes, especially C1 and C3. Still, the IC_50_ value of ~800 µM does not classify them as a group of highly toxic compounds [31], especially since the cholinesterase reactivation or inhibition activities of these oximes were observed at much lower concentrations. Though it would be interesting to determine and understand the mechanism behind this toxicity on the cell molecular level, this oximes scaffold proved to be acceptable for future studies, especially in view of specific cholinesterase inhibitor development. Of course, the difference between in vitro and in vivo translational variations must also be considered for further research [4].

## 3. Materials and Methods

### 3.1. Synthesis of Cinchona Oximes

All reagents and solvents were used as purchased from commercial suppliers without further purification. The reactions were monitored by thin-layer chromatography plates, Silica Gel 60 F_254_ plates (Merck, Darmstadt, Germany). Thin-layer chromatography (TLC) plates were visualized by ultraviolet irradiation (254 nm) or by iodine fumes. Melting points were determined on a Melting Point B-540 apparatus (Büchi, Essen, Germany) and are uncorrected. 1D and 2D, ^1^H- and ^13^C-NMR spectra were recorded at 22 °C with Bruker Avance III HD 400 MHz/54 mm Ascend spectrometer (Bruker Optics Inc, Billerica, MA, USA). Chemical shifts are given in ppm downfield from tetramethylsilane (TMS) as an internal standard and coupling constants (*J*) in Hz. Splitting patterns are designated as s (singlet), d (doublet), dd (doublet of doublets), t (triplet), q (quartet) or m (multiplet). Quinoline hydrogen and carbon atoms are marked with an apostrophe. High resolution mass spectra (HRMS) were obtained on a 4800 plus MALDI TOF/TOF instrument (Applied Biosystems Inc, Foster City, CA, USA).

*Cinchonin-9-one:* Cinchonin-9-one was synthesized according to the published procedure starting from cinchonidine (≥98.0%, Sigma-Aldrich, St. Louis, MO, USA) [32]. After acid-base work up, a yellow solid was obtained. Yield: 48%. m.p. 113–114 °C; IR: 1698 cm^−1^ (C=O); ^1^H-NMR (400 MHz, CDCl_3_-*d*) δ/ppm 1.47–1.63 (1H, m, H7a) 1.63–1.76 (1H, m, H4) 1.82–1.98 (2H, m, H5a, H7b) 2.11–2.31 (1H, m, H5b) 2.31–2.46 (1H, m, H3) 2.57–2.72 (1H, m, H2b) 2.76–2.98 (2H, m, H6) 3.04–3.26 (1H, m H2a) 4.14–4.26 (1H, m, H8) 5.02–5.18 (2H, m, H11) 5.90–6.05 (1H, m, H10) 7.59–7.64 (1H, m, H3′) 7.64–7.72 (1H, m, H7′) 7.77 (1H, ddd, *J* = 8.38, 7.06, 1.51 Hz, H6′) 8.18 (1H, d, *J* = 8.29 Hz, H5′) 8.20–8.29 (1H, m, H8′) 9.02 (1H, t, *J* = 4.14 Hz, H2′); ^13^C-NMR (75 MHz, CDCl_3_-*d*) δ/ppm 21.78 (C7) 26.91 (C5) 27.38 (C4) 39.51 (C3) 49.71 (C6) 55.55 (C2) 63.33 (C8) 114.73 (C11) 119.15 (C3′) 124.51 (C9′) 125.05 (C5′) 127.97 (C7′) 129.64 (C6′) 130.11 (C8′) 141.41 (C10) 143.58 (C4′) 149.11 (C10′) 149.76 (C2′) 202.89 (C=O); MALDI-HRMS (*m/z*): calculated 293.1654 (C_19_H_21_N_2_O + H^+^), found 293.1659.

*Cinchona 9-oxime* (C1). Cinchonidin-9-one (1.7 mmol) and hydroxylamine hydrochloride (3.4 mmol) in ethanol (3 mL) were heated under reflux for 24 h. The solvent was removed under reduced pressure. Yellow oil was dissolved in ice-cold water and pH adjusted to 6.5 with 5 M NaOH. After extraction with ether and evaporating the solvent, Cinchona 9-oxime was purified by column chromatography on alumina with chloroform:methanol = 9:1 as eluent. Off-white solid. Oxime in a solution exist as a mixture of 8-(*S*)- and 8-(*R*)-epimers (*S*:*R* = 1:2.3). Yield: 63%; m.p. 98–99° C. IR: 1634 cm^−1^ (C=N–OH); (*S*)-epimer: ^1^H-NMR (400 MHz, CDCl_3_-*d*) δ/ppm: 1.57 (1 H, d, *J* = 5.46 Hz, H7a) 1.67–1.80 (1H, m, H4) 1.82–1.97 (2H, m, H7b, H5b) 2.05–2.16 (1H, m, H5a) 2.32 (1H, s, H3) 2.60–2.85 (2 H, m, H2) 3.04–3.34 (2H, m, H6) 3.66–3.79 (1H, m, H8) 4.97–5.12 (2H, m, H11) 5.80–5.96 (1H, m, H10) 7.21–7.28 (1H, m, H3′) 7.46–7.63 (1H, m, H7′) 7.65–7.80 (2H, m, H5′, H6′) 8.13–8.23 (1H, m, H8′) 8.87–8.99 (1H, m, H2′); ^13^C-NMR (101 MHz, CDCl_3_-d) δ/ppm: 23.02 (C5) 27.67 (C7) 27.73 (C4) 39.67 (C3) 41.92 (C6) 55.64 (C2) 59.90 (C8) 114.50 (C11) 118.64 (C3′) 119.91 (C7′) 124.68 (C9′) 126.94 (C5′) 129.41 (C6′) 130.09 (C8′) 141.76 (C10) 141.92 (C4′) 148.04 (C10′) 149.91 (C2′) 155.12 (C=N); (*R*)-epimer: ^1^H-NMR (400 MHz, CDCl_3_-*d*) δ/ppm: 1.43–1.54 (1H, m, H5a) 1.59 (2H, d, *J* = 7.81 Hz, H7) 1.81 (1 H, H4) 2.22–2.35 (1 H, m, H3) 2.40 (1 H, dd, *J* = 13.07, 9.17 Hz, H5b) 2.72–2.93 (3 H, m, H2, H6a) 2.93–3.11 (1H, m, H6b) 3.61–3.73 (1H, m, H8) 5.00–5.11 (2H, m, H11) 5.94–6.06 (1H, m, H10) 7.15–7.25 (1 H, m, H3′) 7.47–7.57 (1H, m, H7′) 7.59–7.72 (2H, m, H6′, H5′) 8.12–8.23 ( H, m, H8′) 8.80 (1H, d, *J* = 4.29 Hz, H2′); ^13^C-NMR (101 MHz, CDCl_3_-*d*) δ/ppm: 23.15, 23.77 (C5) 26.44 (C7) 28.03 (C4) 40.14, 40.30 (C3) 48.07, 48.52 (C6) 48.76, 48.93 (C2) 59.68, 60.54 (C8) 114.60 (C11) 118.57 (C3′) 120.22 (C7′) 124.94, 125.63 (C9′) 125.35 (C5′) 126.57, 126.65 (C6′) 129.39, 129.82 (C8′) 140.44, 140.56 (C10) 142.75, 143.71 (C4′) 147.71, 147.78 (C10′) 149.61, 149.77 (C2′) 153.46, 153.97 (C=N); MALDI-HRMS (*m/z*): calculated 308.1763 (C_19_H_22_N_3_O + H^+^), found 308.1751.

*N-Methyl-9-hydroxyiminocinchonium iodide* (C2). A solution of Cinchona 9-oxime (0.31 mmol) and methyl iodide (0.32 mmol) in dry acetone was heated in reflux and reaction was monitored with TLC. Solvent was evaporated under reduced pressure and yellow oil was washed with ether three times. Yellow solid. Yield: 81%; m.p. 140 °C decomp. IR: 1634 cm^−1^ (C=N–OH); ^1^H-NMR (400 MHz, DMSO-*d_6_*) δ/ppm: 2.0–2.1 (5H, m, CH_3_, H7) 2.11–2.25 (1H, m, H4) 2.29–2.42 (1H, m, H5a) 2.83–2.96 (2 H, m, H5b, H3) 3.46–3.73 (2H, m, H2) 3.73–3.89 (1H, m, H6b) 3.97–4.20 (1H, m, H6a) 4.54–4.80 (1H, m H8) 5.11–5.35 (2H, m, H11) 5.81–6.10 (1H, m, H10) 7.53–7.62 (1H, m, H3′) 7.63–7.77 (2H, m, H7′, H6′) 7.79–7.88 (1H, m, H5′) 8.09–8.15 (1H, m, H8′) 9.04(1H, dd, *J* = 4.28, 3.06 Hz, H2′); ^13^C-NMR (101 MHz, DMSO-*d_6_*) δ/ppm: 25.04 (C5) 26.90 (C4) 27.92 (C7) 31.22 (CH_3_) 37.62 (C3) 53.23 (C6) 59.48 (C2) 68.06 (C8) 117.65 (C11) 120.67 (C3′) 124.50 (C9′) 125.26 (C7′) 127.58 (C6′) 128.15 (C5′) 130.26 (C8′) 138.13 (C10) 140.47 (C4′) 148.31 (C10′) 150.42 (C=N) 150.73 (C2); MALDI-HRMS (*m*/*z*): calculated 322.1914 (C_20_H_24_N_3_O^+^), found 322.1918.

*N-Benzyl-9-hydroxyiminocinchonium bromide* (C3). A solution of the Cinchona 9-oxime (0.25 mmol) and benzyl bromide (0.26 mmol) in dry acetone was heated in reflux and the reaction was monitored with TLC. The solvent was evaporated under reduced pressure and orange oil was washed with ether three times. Orange solid. Yield: 69%; m.p. 170 °C decomp. IR: 1634 cm^−1^ (C=N–OH); ^1^H-NMR (400 MHz, DMSO-*d_6_*) δ/ppm: 1.82–1.94 (2H, m, H7) 1.94–2.23 (2H, m, H4, H5a) 2.34–2.39 (1H, m, H5b) 2.72–2.83 (1H, m, H3) 3.43–3.65 (2H, m, H2) 3.81–3.98 (1H, m, H6b) 4.09–4.27 (1H, m, H6a) 4.66–4.80 (1H, m, H8) 4.81–5.09 (2 H, m, CH_2_) 5.19–5.38 (2 H, m, H11) 5.83–6.08 (1H, m, H10) 7.45–7.49 (1H, m, H3′) 7.53–7.65 (5H, m, Ar) 7.72–7.80 (2H, m, H5′, H7′) 7.84–7.98 (1H, m, H6′) 8.06–8.17 (1H, m, H8′), 8.99–9.11 (1H, m, H2′); ^13^C-NMR (101 MHz, DMSO-*d_6_*) δ/ppm: 24.46 (C5) 26.98 (C4) 28.46 (C7) 37.30 (C3) 55.83 (C6) 59.90 (C2) 65.39 (CH_2_) 66.93 (C8) 117.64 (C11) 121.37 (C3′) 124.73 (C9′) 125.73 (C7′) 127.57 (C4*) 128.05 (C6′) 128.11 (C1*) 129.56 (C3*, C5*) 130.27 (C5′) 130.73 (C8′) 134.04 (C2*, C6*) 137.43 (C10) 140.22 (C4′) 148.25 (C10′) 150.73 (C2′) 150.41 (C=N); MALDI-HRMS (*m*/z): calculated 398.2227 (C_26_H_28_N_3_O^+^), found 398.2234.

### 3.2. Quantum Chemical Calculations

All quantum chemical calculations were carried out using Gaussian 09 [33]. Geometry optimizations and harmonic frequency calculations were performed using density functional theory with B3LYP functional and 6-311++G(d,p) basis set. Afterward, magnetic isotropic shieldings were calculated at the same level of the theory. NMR spectra were estimated relative to tetramethylsilane.

### 3.3. Chemicals 

The synthesized Cinchona oximes were dissolved in DMSO as 100 mM. Paraoxon-ethyl was purchased from Sigma-Aldrich, while sarin, cyclosarin, tabun and VX from NC Laboratory (Spiez, Switzerland). Stock solutions of sarin, cyclosarin, tabun and VX (5000 μg/mL) were prepared in isopropyl alcohol and further dilutions were made in water at 10^−5^ M just before use, except paraoxon, which was diluted in ethanol. OPs were used in accordance with safe use and safe disposition of highly toxic compounds. ATCh and thiol reagent 5,5′-dithiobis(2-nitrobenzoic acid) (DTNB) were purchased from Sigma-Aldrich (St. Louis, MO, USA). Stock solutions were prepared in water or 0.1 M sodium phosphate buffer pH 7.4. The final concentration of ATCh in reactivation experiments was 1.0 mM. The final concentration of DTNB was 0.33 mM for all activity measurements at 25 °C. Enzymes, recombinant human AChE and purified human plasma BChE, were a gift from Dr. Florian Nachon (Institut de Recherche Biomédicale des Armées, Brétigny-sur-Orge, France). MTS reagent [3-(4,5-dimethylthiazole-2-yl)-2,5-diphenyl tetrazolium bromide] for cytotoxicity assays was purchased from Promega (Madison, WI, USA).

### 3.4. pK_a_ Determination

Acid dissociation constant (*K*_a_) of the oxime group was determined by measuring the degradation of ATCh (1 mM) by 100 µM oxime (oximolysis) at 412 nm and in pH range 4.4–11.3 (0.1 M phosphate buffer; pH < 9.25 was prepared with 0.2 M NaOH) as described previously (20). p*K*_a_ values were calculated using the modified equation described previously [34]:

(1) $$v=k\cdot \left[\mathrm{ATCh}\right]\cdot \left[\mathrm{oxime}\right]\cdot {\left(\frac{K_{a}}{K_{a}+\left[H^{+}\right]}\right)}^{n}$$

where *v* is the rate of oximolysis, *k* is oximolysis constant and *n* is Hill coefficient. Oximolysis was measured in triplicates and corrected for spontaneous degradation of ATCh and DTNB at different pH.

Since the acid-base equilibrium of other functional groups may interfere with the p*K*_a_ determination from oximolysis (as seen from the Hill coefficients if *n* < 1), p*K*_a_ values were also predicted in silico using Marvin software (version 16.11.7.0, ChemAxon, Budapest, Hungary). In such a way in silico determined p*K*_a_ values of the oxime group will serve as a confirmation of the p*K*_a_ values of the oxime group determined in vitro*.*

### 3.5. Determination of Oxime Inhibition Constants

To determine enzyme-oxime dissociation constant *K*_i_ (the concentration of an oxime at which it inhibits 50% of enzyme activity), we measured the enzymes’ activity in the presence of a wide range of oximes´ concentrations ensuring 20–80% inhibition compared to the control activity. The assay was performed in the 96 well-plates on the Tecan Infinite M200PRO plate reader (Tecan Austria GmbH, Salzburg, Austria). The inhibition mixture (300 µL final) contained a buffer, enzyme (AChE, 0.3 nM or BChE, 2.8 nM), oxime and DTNB (0.33 mM final), and after the addition of ATCh (1.0 mM final), the activity was assayed by Ellman’s method [35]. Each oxime-substrate pair was measured in duplicate. For oximes dissolved in DMSO, the final DMSO concentration was kept under 0.5% to eliminate its influence on enzyme activity. The measured activity was corrected for the oxime-induced hydrolysis of ATCh. The *K*_i_ values were determined from experimental points obtained from at least two experiments using the Prismsoftware (Version 6, Graph Pad Software, San Diego, CA, USA) and were evaluated from the effect of substrate concentration (*s*) on the degree of inhibition according to the Hunter-Downs equation [36]:

(2) $$K_{\mathrm{app}}=\frac{v_{i}\cdot i}{v_{o}-v_{i}}=K_{\left(I\right)}+\frac{K_{\left(I\right)}}{K_{\left(S\right)}}\cdot s$$

where *K*_app_ is the apparent enzyme-oxime dissociation constant at a given substrate concentration (*s*), calculated from the enzyme activities *v*_o_ and *v*_i_ measured in the absence and in the presence of the oxime (*i*), respectively. *K*_(I)_ is the enzyme-oxime dissociation constant of a complex formed in the catalytic site (*K*_i_) or allosteric site constant (*K*_i_′). *K*_(S)_ is the enzyme-substrate dissociation constant corresponding to the Michaelis constant (*K*_M_) or to the substrate inhibition constant (*K*_ss_). The values of *K*_(I)_ and *K*_(S)_ were intercepts of the line on the ordinate and abscissa, respectively. When the plot is non-linear function of *s*, the oxime binds to both the catalytic and peripheral allosteric site. *K*_i_ is then obtained from the initial linear part of the curve at the lower substrate concentrations. Since 1.0 mM ATCh was the highest concentration used, the *K*_i_′ from the higher substrate concentration range was not determined.

### 3.6. Oxime-Assisted Reactivation of OP-Inhibited Cholinesterases

Recombinant human AChE and purified human plasma BChE were incubated with all of the OPs (10^−5^ M) up to 1 h, achieving 95–100% inhibition. The inhibited enzymes were passed through a Strata C18-E column (Phenomenex, Torrance, CA, USA) to remove the excess of unconjugated OP. After filtration, the enzyme was incubated with 0.1 mM oximes (or 0.05 mM oxime C3) and at specified time intervals (up to 24 h), an aliquot was taken and diluted 40-fold in buffer containing DTNB. Enzymes concentration in the reactivation mixture were 0.016 µM for AChE and 0.122 µM for BChE. The recovered enzyme activity was measured upon addition of the substrate ATCh (1 mM) by the Ellman method [35]. More precisely, at least 10 experimental points of reactivation were obtained within 24 h. An equivalent sample of the uninhibited enzyme was passed through a parallel column, diluted to the same extent as the inhibition mixture, and control activity was measured in the presence of the oxime at concentrations used for reactivation. Both the activities of the control and the reactivation mixture were corrected for oxime-induced hydrolysis of ATCh. No spontaneous reactivation occurred. Enzyme activity measurements were performed at 25 °C and 412 nm, on the CARY 300 spectrophotometer (Varian Inc., Mulgrave, Australia) with a temperature controller. The reactivation screening was done at a given oxime concentration wherefrom the observed first-order reactivation rate constant (*k*_obs_) and maximal reactivation (React_max_) were determined as previously described [17] from experimental points obtained from at least two experiments using the Prism software (Version 6, Graph Pad Software, San Diego, CA, USA).

### 3.7. Cytotoxicity Screening

Cells for oxime cytotoxicity profiling were procured from the American Type Culture Collection (Rockville, MD, USA): Human Caucasian hepatocyte carcinoma (HepG2, ATCC HB8065) and from European Collection of Authenticated Cell Cultures (Salisbury, England, UK): Human neuroblastoma (SH-SY5Y, ECACC 94030304). SH-SY5Y were grown in Dulbecco’s modified Eagle’s medium F12 (DMEM, Sigma-Aldrich, Steinheim, Germany) supplemented with 15% fetal bovine serum (Gibco, Paisley, UK), 2 mM glutamine, 1% non-essential amino acids and HepG2 were grown in Eagle’s Minimum Essential Medium with Earle′s salts and sodium bicarbonate, without l-glutamine (EMEM) supplemented with 10% (*v/v*) fetal bovine serum (Gibco, Paisley, UK) at 37 °C in a 5% CO_2_ atmosphere, according to the manufacturer’s instructions. Cells were detached with 0.25% Trypsin/EDTA solution (Sigma-Aldrich, St. Louis, MO, USA), re-suspended and seeded at 96-well for experiments. Cells were treated and exposed for 24 h to Cinchona oximes (6.25–800 μM). Cytotoxicity was determined using the standard MTS detection reagent assay measuring the succinate dehydrogenase mitochondrial activity of living cells [37]. Procedure followed a previously described protocol [24]. Cells and oximes were incubated for 24 h at 37 °C. 120 µL of MTS mix reagent (CellTiter 96 AQueous One Solution Cell Proliferation Assay, Promega, Madison, WI, USA) was added in each well incubated 0.5–3 h (different cell lines) after which the absorbance was read at 492 nm on a Tecan Infinite M200PRO plate reader. Total percentage of DMSO in cytotoxicity assay was 0.8%. The assay was performed in 96-well in 120 µL/well media volume with 100,000 or 400,000 cells. Data was evaluated using predefined IC_50_ equation from the Graph Pad Prism programme.

## 4. Conclusions

We evaluated a new Cinchona structure scaffold as a potential way forward in the development of more efficient treatment in OP poisoning. Though, they were poor AChE reactivators they showed somewhat promising results in BChE reactivation in terms of their observed universal activity against various OPs. Furthermore, they were shown to be a good starting structure for development of selective ligands with high affinity for binding to the active site of BChE. Future studies of such compounds were also supported by cytotoxicity results as long as their biological activity was targeted in the lower micromolar range.

## Figures and Tables

**Table 1:** Screening of OP-inhibited hBChE reactivation by 0.1 mM oximes C1, C2 and 0.05 mM oxime C3. Observed reactivation rate constants (*k*_obs_), maximal reactivation (*React*_max_) obtained in time (*t*) are presented as the mean value of two experiments.

OP	Oxime	*k*_obs_ (min^−1^)	*React_max_* (%)	*t* (h)
VX	C1	-	20	24
	C2	0.0064	60	24
	C3	-	15	22
	HI-6	0.0356	85	2.5
Sarin	C1	-	10	24
	C2	0.0024	70	24
	C3	0.0010	65	22
	HI-6	0.0113	98	24
Cyclosarin	C1	0.0008	45	24
	C2	0.0040	45	24
	C3	-	35	22
	HI-6	0.0260	70	24
Paraoxon	C1	-	10	24
	C2	-	30	24
	C3	-	15	22
	Obidoxime	0.0020	60	24
Tabun	C1	-	10	24
	C2	0.0015	60	24
	C3	-	15	22
	K117	0.0095	67	24

**Table 2:** Reversible inhibition of hAChE and hBChE by the tested oximes. Dissociation inhibition constants (*K*_i_) was evaluated from at least three experiments studied in 0.3–1.0 mM substrate ATCh concentration range.

Oxime	Concentration (µM)	hAChE	hBChE	hAChE/hBChE
		*K*_i_ (µM) ± S.E.		
C1	100–400	432 ± 52	-	272
	1–400	-	1.59 ± 0.06	
C2	100–400	323 ± 77	-	29.9
	10–400	-	10.8 ± 0.99	
C3	15–100	3.72 ± 0.49	-	5.8
	0.5–25	-	0.64 ± 0.08	

## Supplementary Materials

The following are available online, Figure S1: Time-dependent course of reactivation of hBChE, Figure S2: Atom numbering of Cinchona alkaloid derivatives, Figures S3–S14: IR, 1H and 13C NMR spectra of prepared compounds, Tables S1–S3: Quantum-chemical calculations.
