# Supplementary material for: New Cinchona Oximes Evaluated as Reactivators of Acetylcholinesterase and Butyrylcholinesterase Inhibited by Organophosphorus Compounds
Source: Molecules. 2017 Jul 22;22(7):1234. doi: 10.3390/molecules22071234 (PMC6151989; doi:10.3390/molecules22071234)
Supplement: Supplementary file 1 [file molecules-22-01234-s001.pdf]

Supplemental Material

New Cinchona Oximes Evaluated as Reactivators of Acetylcholinesterase and Butyrylcholinesterase Inhibited by Organophosphorus Compounds

Maja Katalinić [1], Antonio Zandona [1], Alma Ramić [2], Tamara Zorbaz [1], Ines Primožič [2,*] and Zrinka Kovarik [1,*]

[1] Institute for Medical Research and Occupational Health, POB 291, HR-10001 Zagreb, Croatia; mkatalinic@imi.hr (M.K.); azandona@imi.hr (A.Z.); tzorbaz@imi.hr (T.Z.)

[2] Department of Chemistry, Faculty of Science, University of Zagreb, HR-10001 Zagreb, Croatia; alma.ramic@gmail.com

* Correspondence: ines.primozic@chem.pmf.hr (I.P.); zkovarik@imi.hr (Z.K.); Tel.: +385-1-4606-408 (I.P.); +385-1-4682-555 (Z.K.)

Content

S1. Time-dependent course of reactivation of hBChE 2

S2. Atom numbering of Cinchona alkaloid derivatives 3

S3. IR, ^{1}H and ^{13}C NMR spectra of prepared compounds 4

Cinchonin-9-one 4

Cinchona-9-oxime (**C1**) 5

N-methyl-9-hydroxyiminocinchonium iodide (**C2**) 7

N-benzyl-9-hydroxyiminocinchonium bromide (**C3**) 8

S4. Quantum-chemical calculations 10

S1. Time-dependent course of reactivation of hBChE

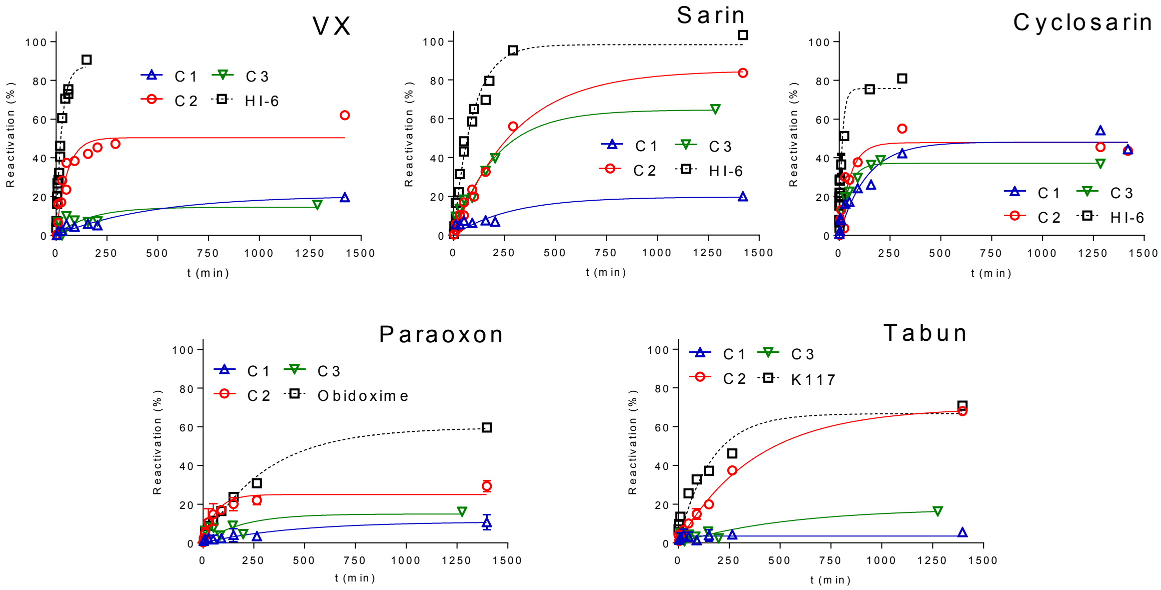

Figure S1. Time-dependent course of reactivation of hBChE by 0.1 mM oximes (or 0.05 mM C3) at 25 °C.

S2. Atom numbering of Cinchona alkaloid derivatives

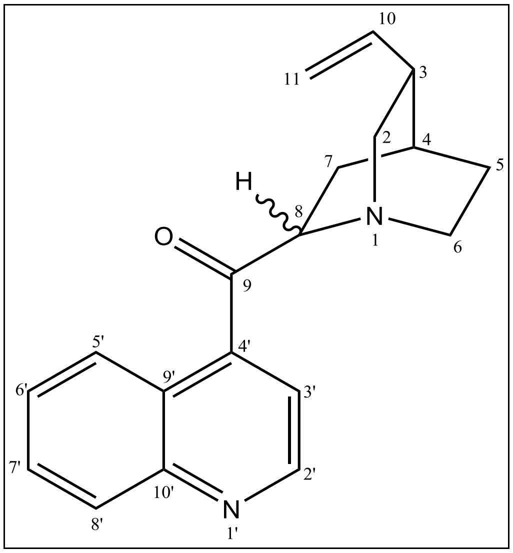

Figure S2. Labeling scheme of prepared compounds

S3. IR, ^{1}H and ^{13}C NMR spectra of prepared compounds

Cinchonin-9-one

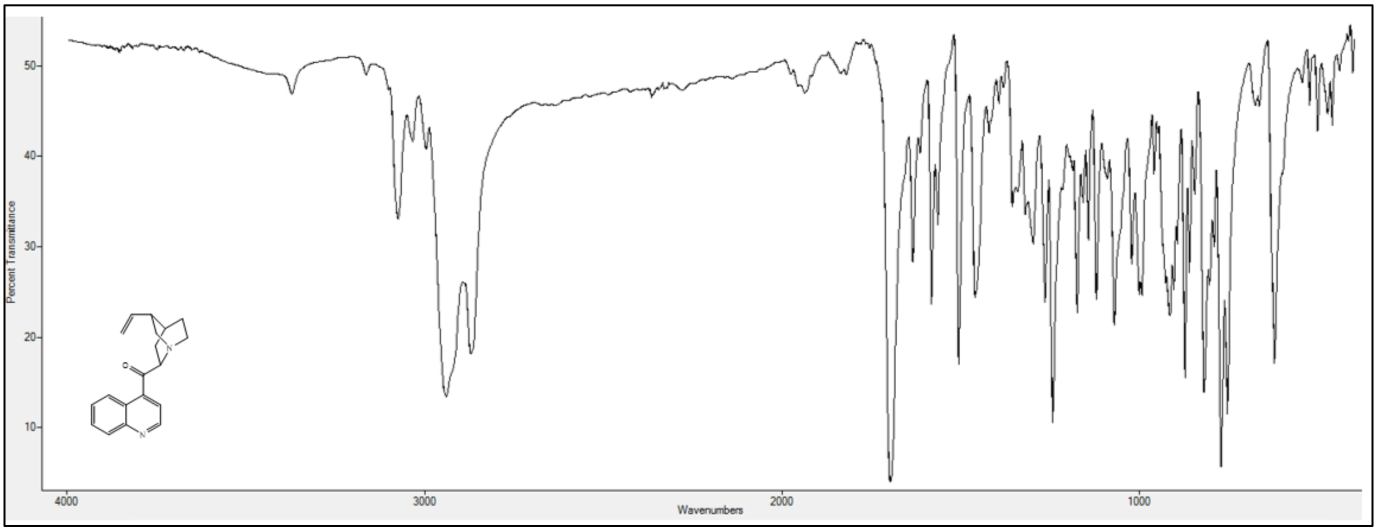

Figure S3. IR spektra of Cinchonin-9-one

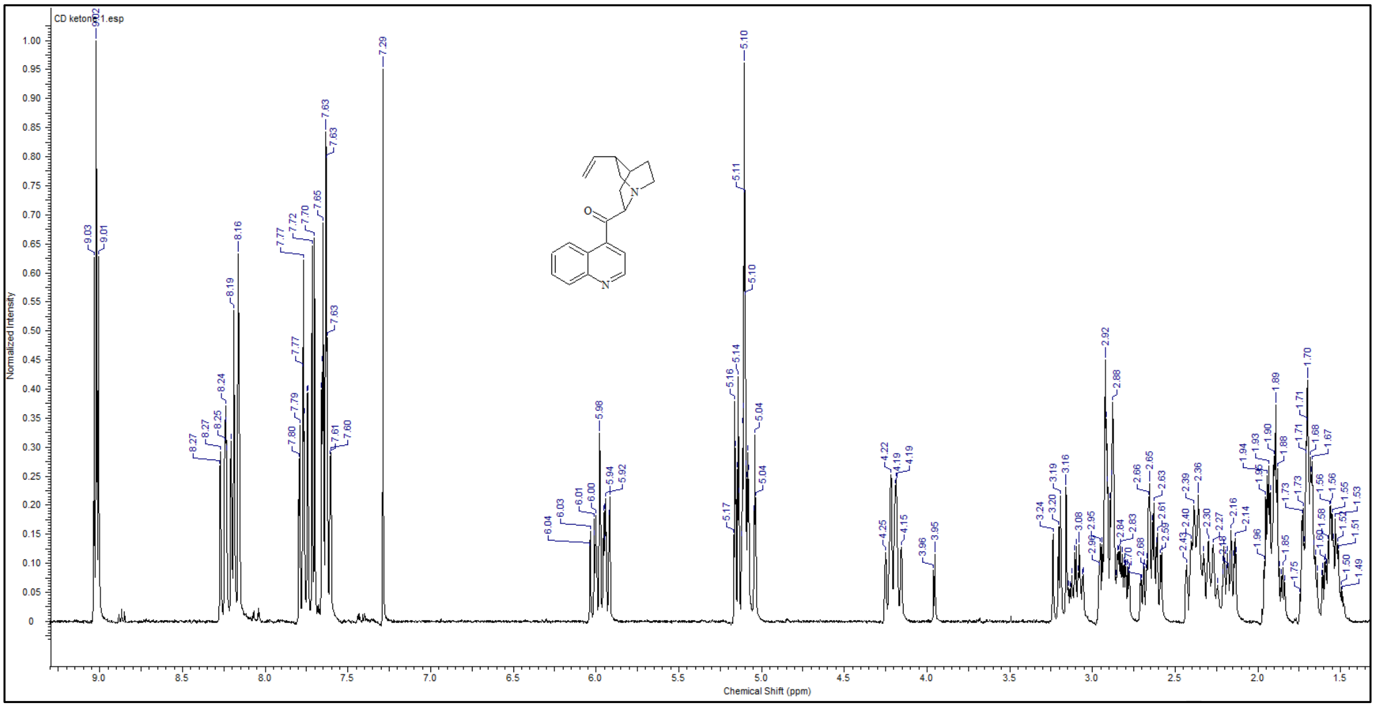

Figure S4. ^{1}H NMR spectra of Cinchonin-9-one

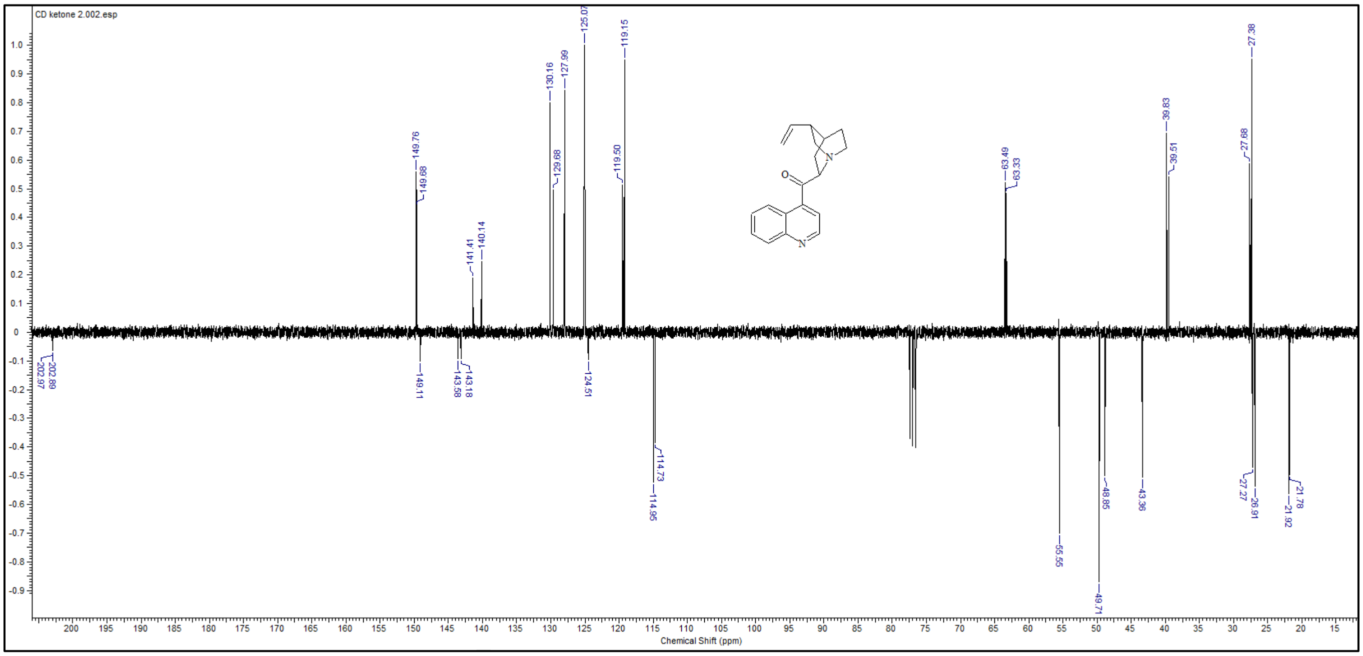

Figure S5. ^{13}C NMR spectra of Cinchonin-9-one

Cinchona-9-oxime (**C1**)

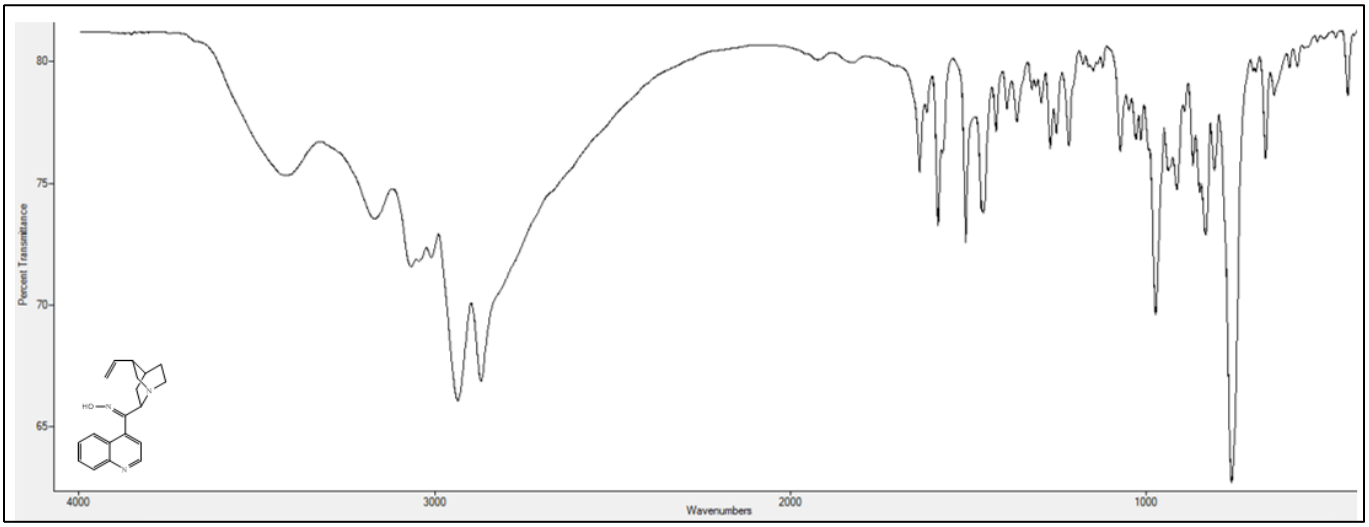

Figure S6. IR spektra of Cinchona-9-oxime

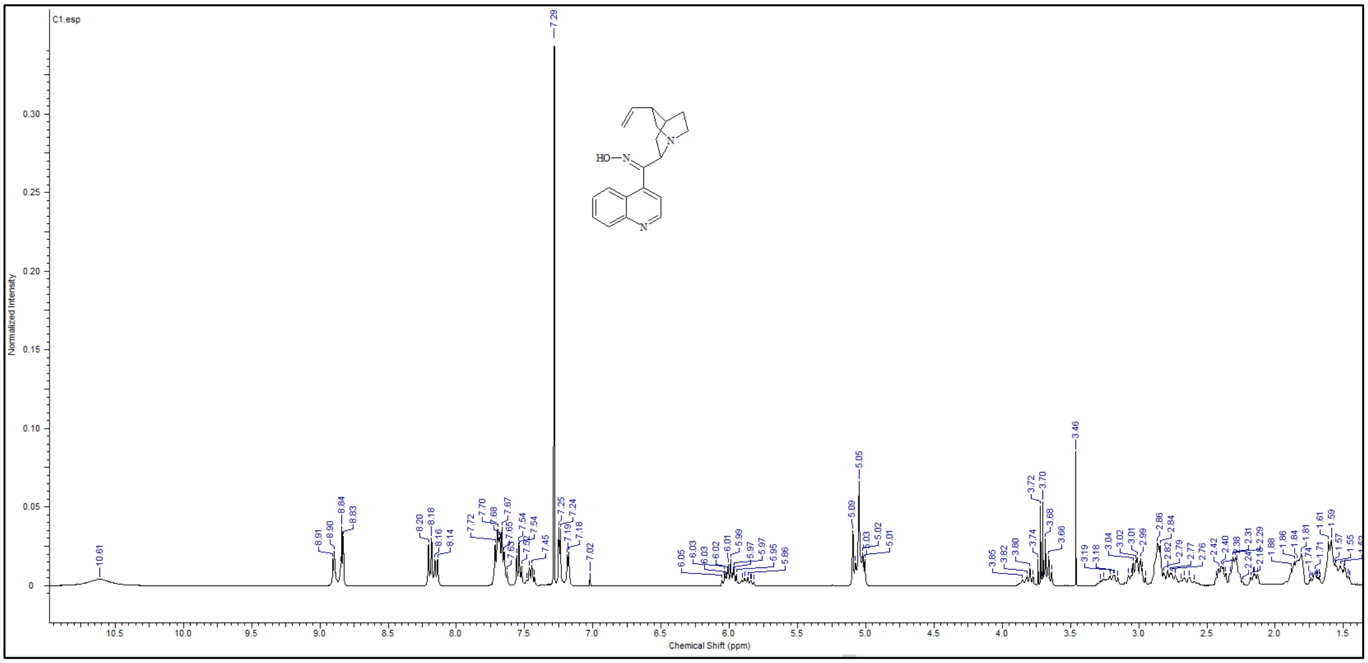

Figure S7. ^{1}H NMR spectra of Cinchona-9-oxime

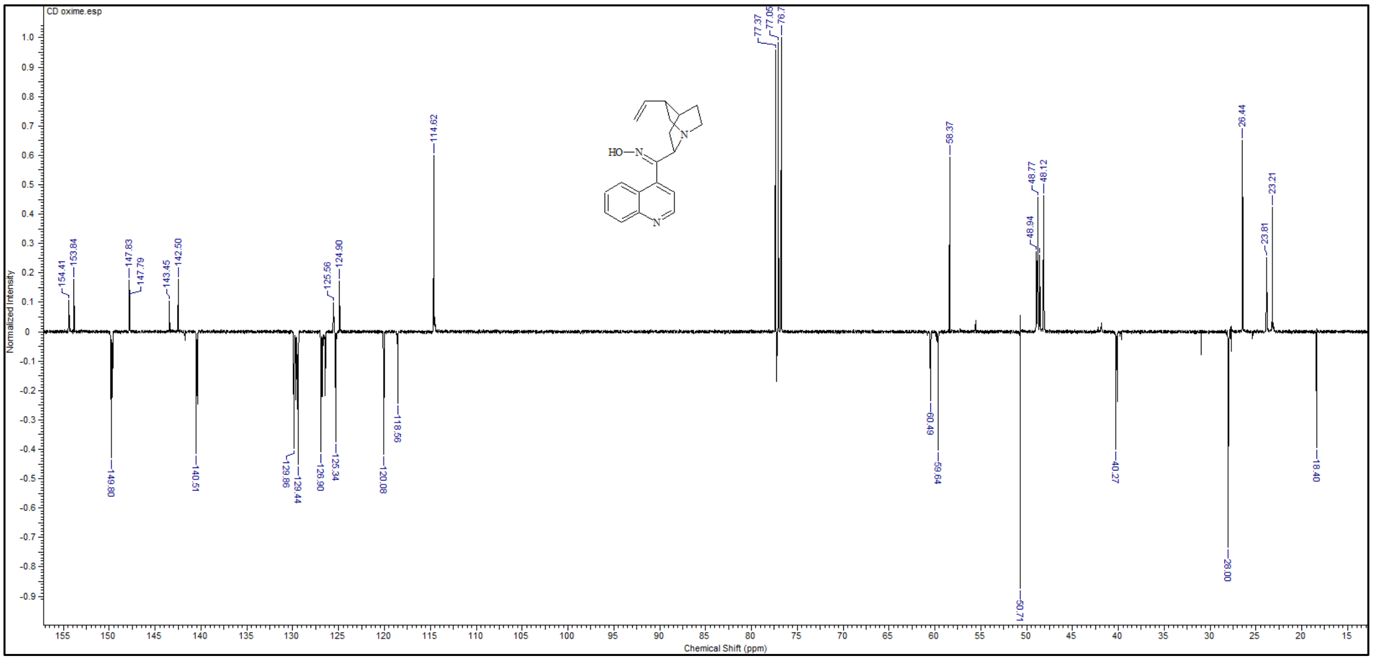

Figure S8. ^{13}C NMR spectra of Cinchona-9-oxime

N-methyl-9-hydroxyiminocinchonium iodide (**C2**)

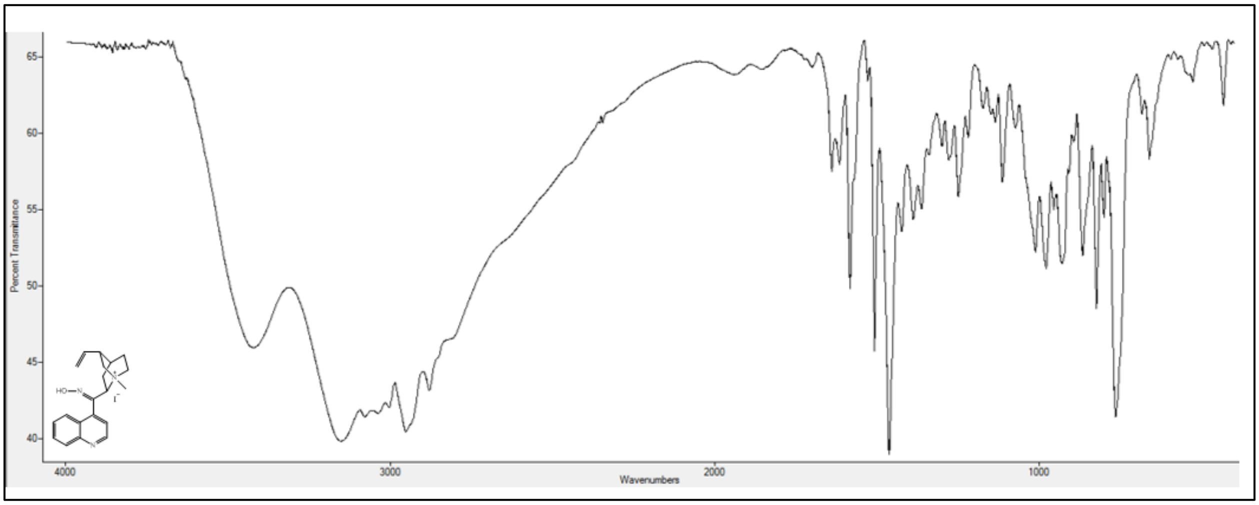

Figure S9. IR spektra of *N*-methyl-9-hydroxyiminocinchonium iodide

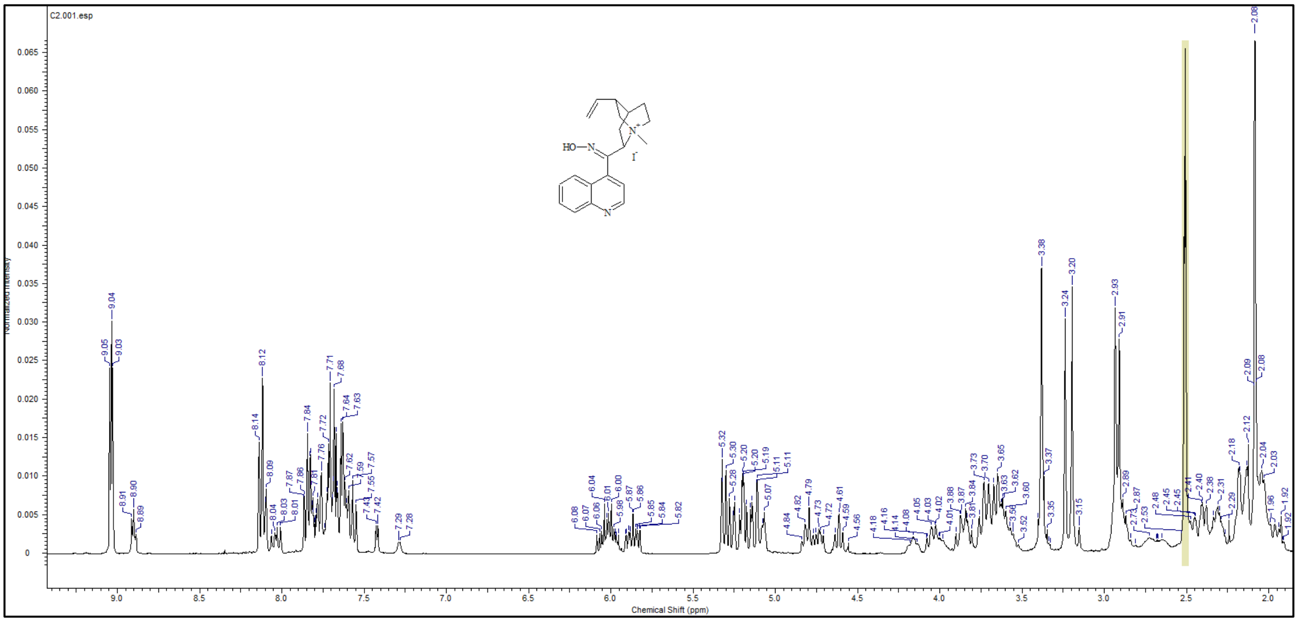

Figure S10. ^{1}H NMR spectra of *N*-methyl-9-hydroxyiminocinchonium iodide

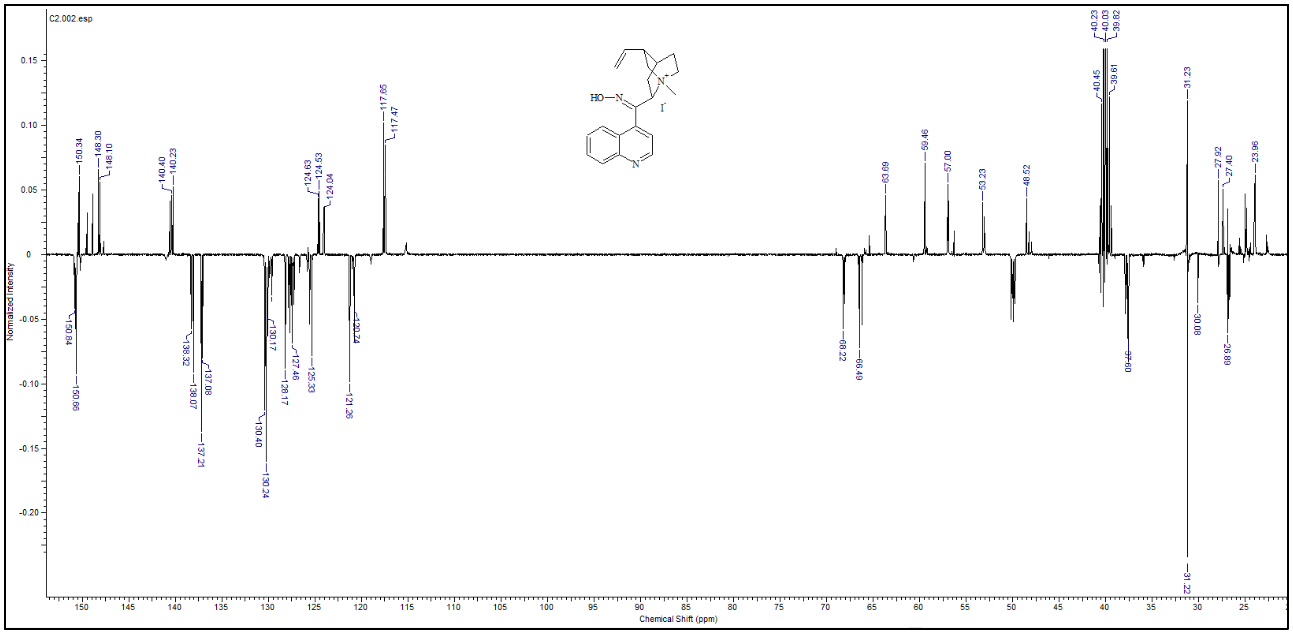

Figure S11. ^{13}C NMR spectra of *N*-methyl-9-hydroxyiminocinchonium iodide

N-benzyl-9-hydroxyiminocinchonium bromide (**C3**)

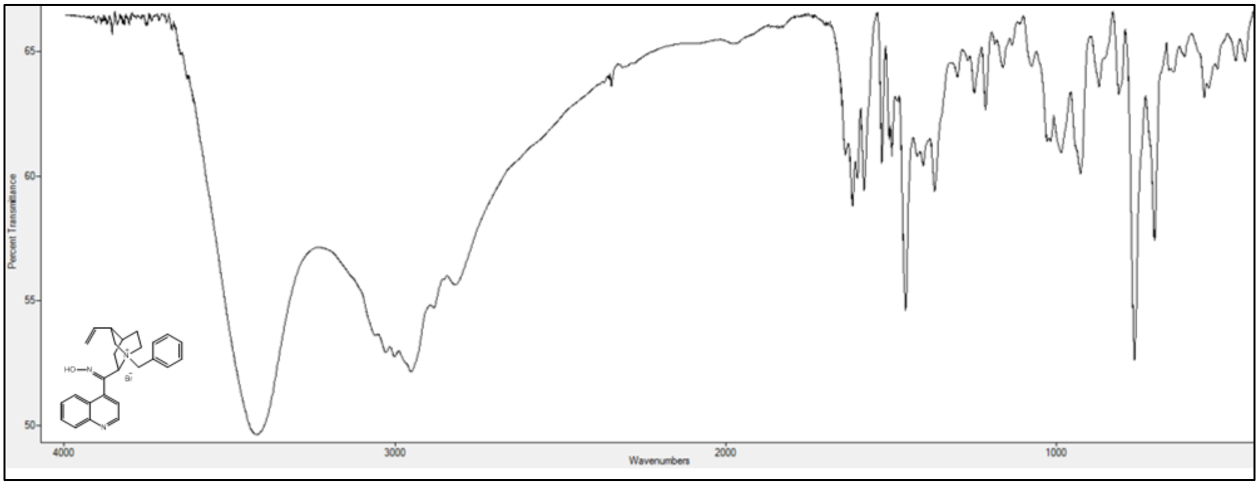

Figure S12. IR spektra of *N*-benzyl-9-hydroxyiminocinchonium bromide

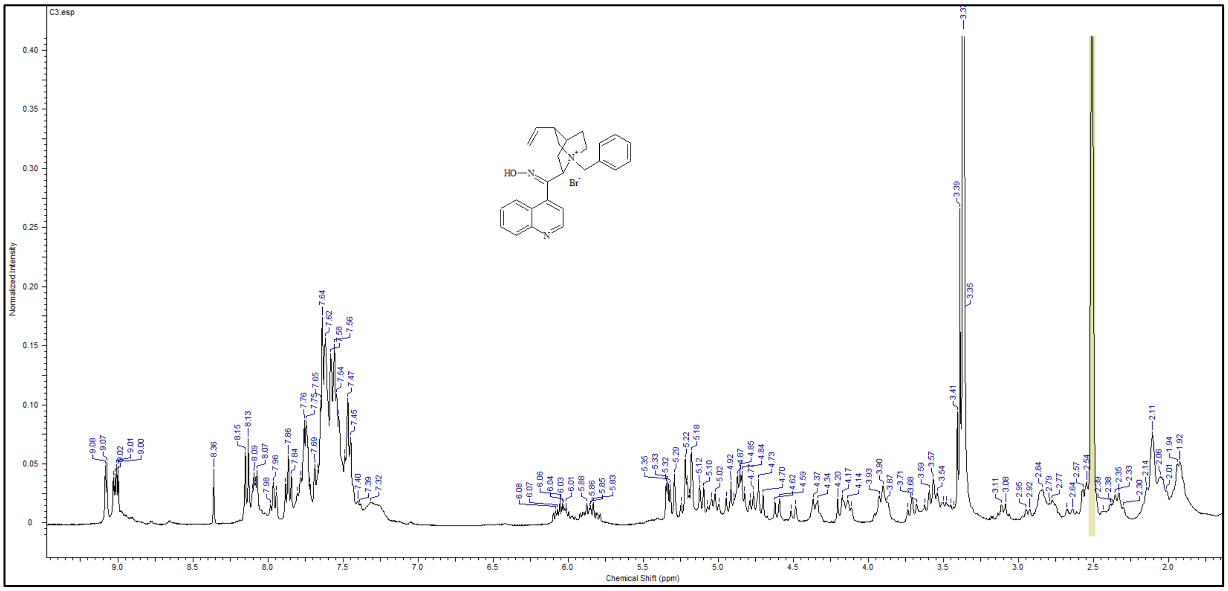

Figure S13. ^{1}H NMR spectra of *N*-benzyl-9-hydroxyiminocinchonium bromide

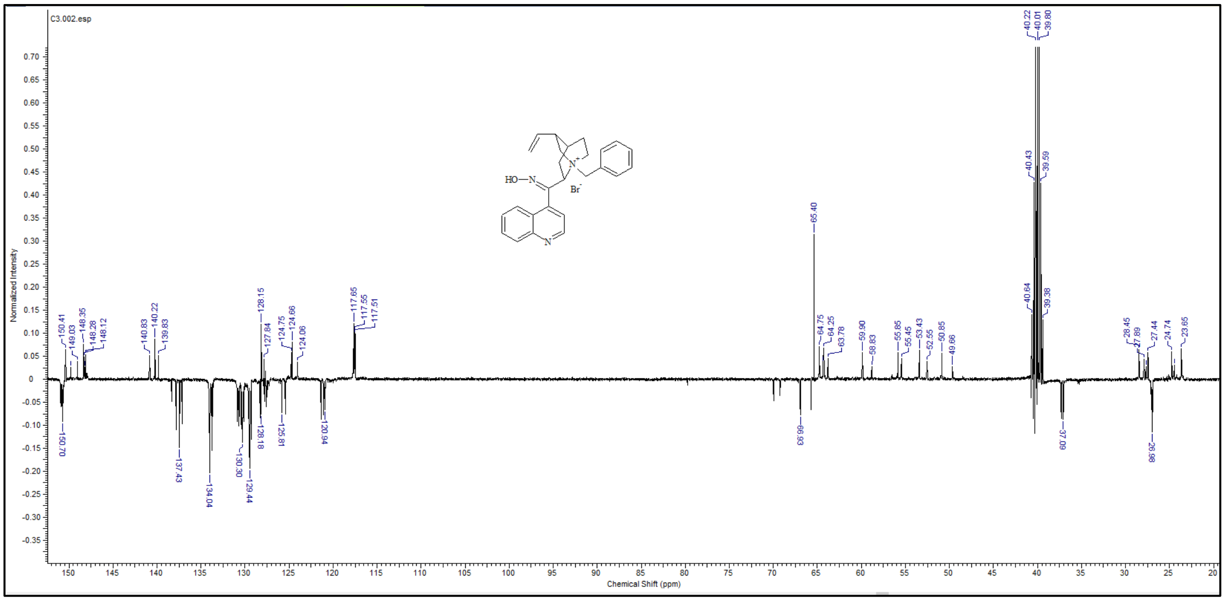

Figure S14. ^{13}C NMR spectra of *N*-benzyl-9-hydroxyiminocinchonium bromide

S4. Quantum-chemical calculations

Table S1. Calculated ^{13}C and ^{1}H NMR chemical shifts (B3LYP/6-311++G(d,p)) for Cinchonin-9-one and Cinchonidin-9-one relative to tetramethylsilane

Atom	δ (Cinchonin-9-one) / ppm	δ (Cinchonidin-9-one) / ppm
C8	67.2	68.4
C6	54.4	45.6
C7	30.2	28.8
C5	32.2	32.8
C4	37.0	36.1
C2	54.5	61.9
C3	49.9	49.2
C9	209.8	215.8
C4'	150.0	155.6
C9'	132.3	131.0
C10'	157.9	157.5
C2'	155.8	155.8
C3'	123.5	126.5
C5'	132.5	134.0
C6'	134.1	133.7
C7'	135.5	135.1
C8'	138.5	138.8
C10	151.0	152.7
C11	119.8	119.9
H8	4.4	4.1
H6	3.0	2.7
H6	3.1	3.6
H7	1.4	2.0
H7	1.6	1.7
H5	1.6	1.5
H5	1.6	2.0
H4	1.5	1.7
H2	3.7	2.8
H2	3.0	3.3
H3	2.1	2.2
H2'	9.3	9.3
H3'	7.5	7.6
H5'	8.9	9.7
H6'	7.9	7.8
H7'	8.0	8.0
H8'	8.5	8.6
H10	6.3	6.2
H11	5.1	5.2
H11	5.3	5.3

Table S2. Calculated ^{13}C and ^{1}H NMR chemical shifts (B3LYP/6-311++G(d,p)) for *anti* and *syn* cinchonin-9-on ketoxime relative to tetramethylsilane

Atom	δ (*anti*-cinchonin-9-on ketoxime) / ppm	δ (*syn*-cinchonin-9-on ketoxime) / ppm
C8	69.2	67.4
C6	54.0	54.6
C7	30.4	31.0
C5	32.0	31.7
C4	36.5	36.1
C2	58.1	54.8
C3	49.6	50.3
C9	164.2	165.1
C4'	152.4	150.0
C9'	134.8	132.7
C10'	157.6	156.7
C2'	155.7	156.0
C3'	124.5	123.9
C5'	132.2	133.7
C6'	132.3	132.2
C7'	134.9	134.9
C8'	138.6	138.8
C10	151.2	151.4
C11	119.4	119.4
H8	4.2	3.9
H6	3.0	2.9
H6	3.1	3.0
H7	1.0	1.1
H7	1.5	1.7
H5	1.5	1.5
H5	1.5	1.5
H4	1.3	1.4
H2	3.4	3.4
H2	3.2	2.9
H3	2.1	2.1
H2'	9.2	9.2
H3'	7.3	7.3
H5'	8.6	8.2
H6'	7.8	7.8
H7'	8.0	8.0
H8'	8.5	8.5
H10	6.4	6.4
H11	5.2	5.2
H11	5.3	5.3
OH	6.7	6.3

Table S3. Calculated ^{13}C and ^{1}H NMR chemical shifts (B3LYP/6-311++G(d,p)) for *anti* and *syn* cinchonidin-9-on ketoxime relative to tetramethylsilane

Atom	δ (*anti*-cinchonidin-9-on ketoxime) / ppm	δ (*anti*-cinchonidin-9-on ketoxime) / ppm
C8	65.0	65.9
C6	45.9	47.3
C7	29.7	33.6
C5	32.3	33.5
C4	34.6	36.6
C2	59.7	63.9
C3	49.3	49.8
C9	162.5	168.8
C4'	153.3	149.2
C9'	134.1	132.2
C10'	156.8	157.1
C2'	157.1	155.6
C3'	127.7	125.9
C5'	131.2	131.2
C6'	132.4	132.9
C7'	134.6	135.2
C8'	139.3	138.9
C10	151.4	153.0
C11	121.1	119.3
H8	4.6	3.7
H6	2.7	2.7
H6	3.1	4.0
H7	0.8	1.4
H7	1.8	1.8
H5	1.4	1.4
H5	1.4	1.6
H4	1.3	1.6
H2	2.8	2.6
H2	3.2	3.2
H3	2.1	2.0
H2'	9.3	9.2
H3'	7.5	7.3
H5'	7.8	8.1
H6'	7.8	7.8
H7'	7.9	8.0
H8'	8.5	8.6
H10	6.3	6.0
H11	5.2	5.0
H11	5.3	5.2
OH	13.3	6.3
